# Stochastic Power Consumption Model of Wireless Transceivers †

**DOI:** 10.3390/s20174704

**Published:** 2020-08-20

**Authors:** Paweł Kryszkiewicz, Adrian Kliks, Łukasz Kułacz, Bartosz Bossy

**Affiliations:** Institute of Radiocommunications, Poznan University of Technology, 61-131 Poznan, Poland; adrian.kliks@put.poznan.pl (A.K.); lukasz.kulacz@put.poznan.pl (Ł.K.); bartosz.bossy@put.poznan.pl (B.B.)

**Keywords:** power consumption, wireless transceivers, system level simulations, hardware measurements

## Abstract

Energy efficiency is a key aspect when designing and optimizing contemporary wireless networks and transceivers. Assessment of energy efficiency requires proper energy consumption models. The most common solutions are to measure a single device and propose a device-specific model or to propose a simplified model for many transceivers but not reflecting all phenomena visible in a given transceiver energy consumption. Therefore, it has to be selected to accurately model a single transceiver or coarsely model a wide group of transceivers. This paper proposes a new approach, where a fixed energy consumption model is used but with parameters being random variables. This reflects variability between various transceivers from various vendors. First the model parameters are adjusted separately for each of 14 measured WiFi modems. These devices are treated as samples of a wider population of devices and their parameters are used for stochastic parameters modeling, i.e., choosing the random variables’ distributions, their parameters, and the correlation among parameters. The proposed model can be used, e.g., for system-level network design where variability among transceivers power consumption can be used as a new degree of freedom. The paper presents simulation results for a simple multi-hop link whose energy consumption is characterized in much more detail thanks to the proposed stochastic power consumption model.

## 1. Introduction

The energy efficiency paradigm is gaining importance in the context of wireless communications and networks. Care of the environment on the hand, and the reduction of the operating expenditures by mobile network operators on the other hand, are complemented by common concerns, such as the growing demand for throughput and the fact that an increasing number of services offered are limited by battery capacity of mobile terminals. There are several ways of increasing network energy efficiency, ranging from typical resource allocation optimization [1] to more fundamental changes, e.g., following the network design paradigms observed in the human brain [2]. In order to correctly reflect the operation of the real network in terms of achieved energy efficiency, accurate models representing both the propagation environment and the devices themselves (including energy consumption) are needed. One of the key requirements in the design of energy-efficient communication systems is the estimation of power consumption in the network. In the literature, it is common to observe the utilization of simplistic power consumption models, e.g., a fixed and identical power consumption of transmitter and receiver [3].

There are various approaches to power consumption modeling. One can identify three main types of power consumption models: (i) a high-level analytical model, (ii) a model that is the sum of the analytical models of the transmitter and receiver components, and finally (iii) a power consumption model that is based on measurements. The common disadvantage of all existing methods is that their accuracy and applicability are limited to only a single technological solution. If another architecture (or even the same architecture but another implementation from a different vendor) is to be used, the utilized model, or at least its parameters, might no longer be valid. Moreover, when one wants to assess the network energy consumption, it is necessary to consider a current, often rich, set of utilized transceivers in this network. There are currently two approaches to create such a model.

The first approach involves modeling the energy consumption of all separate components of wireless modules, such as an amplifier or an analog-to-digital converter or mixer, and then adding up the energy consumed by each of them. Unfortunately, this solution would require a very detailed knowledge of applied architecture and of all used modules. Most often it is not possible to access such information, and additionally, in the production of electronic systems, large variations in parameters between individual units can be observed.

The second approach in modeling of network energy consumption involves measuring each utilized transceiver power consumption separately. However, this is impossible to achieve, especially for large networks.

In this work, we propose a new approach. First, a model of energy consumption of a wireless transceiver is created that reflects a variability of implementations. It is parametrized by a set of measurements of WiFi cards (connected via USB). Checking more than a dozen of devices in different propagation conditions and different data rates allowed us to find the model coefficients for each of them. However, one may observe that the model parameters vary significantly among transceivers. This motivates us to construct a stochastic energy consumption model where each model coefficient is a random variable of a specific distribution and its parameters. For better reliability, correlations among model parameters are considered. Finally, a new stochastic transceiver power consumption model is presented and an initial verification of its usefulness in a multihop network is presented. The main novelty of the proposed model comes from the randomness of each device model’s coefficients, which allows us to characterize a whole population of wireless transceivers and a whole network energy consumption. The resultant network power consumption is much more detailed and easier to obtain than in two contemporary utilized approaches described above. This manuscript is an extension of a conference paper [4] in which the authors presented a deterministic power consumption model for each of the considered transceivers. However, all important measurements and data modeling phases are presented in this manuscript as well for completeness of the presentation. The concept of stochastic modeling along with mathematical derivations and validation using simulations are exclusively presented in this paper.

The paper is structured as follows, first, an overview of existing power consumption models is presented. Next, the measurement setup is discussed, followed by the proposal of the new approach to power consumption modeling. In the consecutive chapter, a stochastic power consumption model is derived. Finally, the verification of the proposed model is made, followed by the drawn conclusions.

## 2. Related Works

The estimation of power consumption in the network is a crucial aspect in designing energy-efficient wireless communication systems. The main difficulty in the estimation of power consumption results from different types of technologies, standards, implementations, etc. As we mentioned above, in the literature three approaches to power consumption modeling can be distinguished: (i) a high-level analytical model, (ii) the estimation of the power consumed by each transmitter and receiver components, and (iii) estimating power consumption based on the measurements. The first approach has been applied in [5,6], where the authors considered a model consisting of the constant circuit power and the transmit power allocated at each subchannel. The former component includes the power consumed by the active (i.e., turned on) circuits of transmitters and receivers, whereas the transmit power was assumed to change dynamically according to varying channel conditions and modulation schemes. The constant circuits’ power does not accurately reflect many effects, e.g., signal processing power varying with bitrate. In [7,8,9], circuit power was modeled as the linear function of the achieved data rate. This resulted from the fact that the power dissipation in a chip was modeled as the sum of a static term and dynamic term depending on the clock frequency, which was dynamically scaled with the data rate. These high-level power consumption models, thanks to their simplicity, are commonly used in the design of energy-efficient resource allocation algorithms. However, their disadvantage is low accuracy. In addition, the same power consumption model is used for each transceiver while considering network energy consumption. These models can be outperformed by the stochastic power consumption model proposed in this paper.

The second approach for power consumption modeling known from the literature focuses on the estimation of the power consumed by transmitter and receiver components. For example, the authors in [10,11,12,13] assumed that the total power used in the transmission is the sum of power consumed by the power amplifier (PA), the low noise amplifier (LNA), the analog-to-digital converter (ADC), and the applied signal decoders. A similar approach was also proposed in [14], where the considered components for power consumption modeling included ADC, a digital-to-analog converter (DCA), reconstruction and anti-aliasing filters, mixers, a frequency synthesizer, PA, LNA, and a baseband amplifier. The effects of signal bandwidth, peak-to-average power ratio (PAPR), modulation order, transmission distance, and the signal center frequency on the Radio Frequency (RF) front-end energy consumption were considered and the exemplary power consumption values from the most commonly used designs were provided. In [15], some power consumption values were listed in the context of long term evolution (LTE) technology. As for the encoding/decoding power, [16,17] revealed the number of operations needed to encode or decode the information bit for the common channel coding algorithms. If the information on the energy consumption per operation is known for a given processor, then the total power consumed by channel coding can be assessed. In [18,19,20], the power consumption of baseband processing was estimated using Xilinx software toolboxes and, based on that, the analytical model was proposed. This approach guarantees high accuracy of power estimation, but is highly dependent on implementation, technology, and so on. While the resultant mathematical formulation can be quite complex, some components are negligible in comparison to others. Moreover, this model does not reflect the variability of power consumption among the transceivers used in the network. Our proposed stochastic power consumption model outperforms this model in these areas.

The last approach to estimate the power consumption of the wireless devices is to model it based on the measurements. In [21,22,23], the authors measured the power consumption of a set of commercial devices in the number of configurations. In [21], the power consumption behavior of the 802.11n and 802.11g access points was investigated and compared. The authors observed a similar power consumption pattern for both technologies, namely a linear behavior until the access point reached a saturation point. However, there were relevant differences in the amount of (i) power consumed for transmitting and receiving data and (ii) the traffic rate at which the access point saturated. In [22], a simple first-order polynomial model for the energy utilization of a set of commercially, contemporary wireless transceivers in the context of various communication standards, such as UMTS, LTE, WiFi, and Bluetooth, was proposed and discussed. In [23] power consumption of GSM and UMTS base stations has been measured. A first-order power consumption model in relation to the offered traffic was proposed. For this group of solutions the estimated power consumption model was realistic but only fit the measured device, and thus can be wrong for other technologies (e.g., LTE and IEEE 802.11) or even different designs (e.g., another vendor). Moreover, in some cases (e.g., smartphones) the measurements of the power consumed by wireless communication components can be difficult or even impossible to be carried out. The mathematical simplicity and accuracy of the model and ability to model a whole range of transceivers coming from various vendors is provided by the stochastic power consumption model proposed in this paper.

The last group of solutions is a mix of the approaches described above. For example in [24,25], the first two approaches were applied in a multi-user massive multiple-input and multiple-output (MIMO) scenario and the power consumption of elements specific to massive MIMO processing, such as the channel estimation, the load-dependent backhaul, and linear processing on the base station (e.g., zero-forcing) were modeled, separately. Although some elements were estimated in a high-level manner, some parameters were still dependent on the hardware implementation or constrained by a specific technologies or methods, e.g., power consumption of a decoder only in the case of LDPC codes.

## 3. Measurements Setup

**Figure 1:**
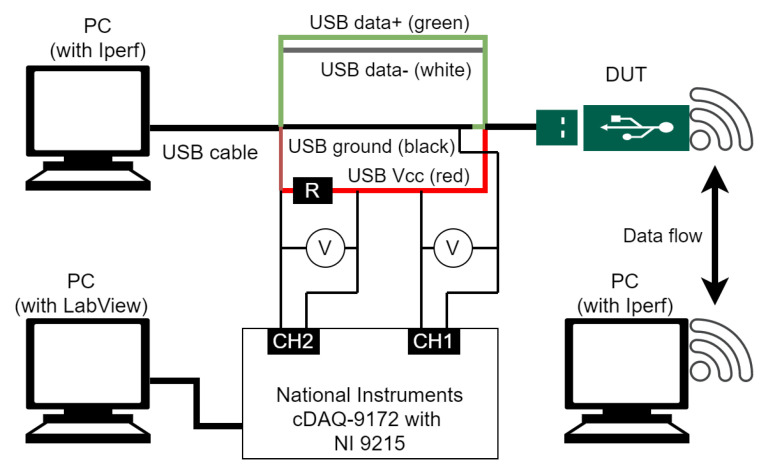
Measurement setup (DUT—Device Under Test).

We first conducted the measurements of power consumption by using a number of off-the-shelf devices, which we used to define the generic power consumption model. The experiment stand shown in Figure 1 was built to measure the power consumed by each individual wireless transceiver. Three computers were used; two connected wirelessly through the measured WiFi dongle (IEEE 802.11g standard), and the third one used to measure power consumption of the tested dongle. The dongle was connected to a computer via a USB cable with a measurement bridge that allowed to directly measure voltage delivered to the USB stick (Channel 1—CH1 in Figure 1). Moreover, observation of the voltage on the shunt resistor of a known resistance (in this case 4.7 Ohms, denoted in the figure as *R*) allowed for the derivation of the current flowing in the WiFi dongle (Channel 2—CH2 in Figure 1). The National Instruments card NI 9215 was used to collect all measurements, and it was connected to a computer with LabView software operating on it. The traffic in the network was generated using Iperf software, allowing the setting of a precisely specified throughput in a network layer (using the UDP protocol). All measured devices are listed in Table 1 along with the utilized WiFi chipset. Please note that some devices from different vendors are based on the same chipset. We measured the power consumption as a function of average rate and distance/pathloss in both WiFi dongle states, i.e., transmitting or receiving data. We also tested the power consumption in idle (USB dongle connected, no wireless traffic) and associated states (modem connected to the network).

**Figure 2:**
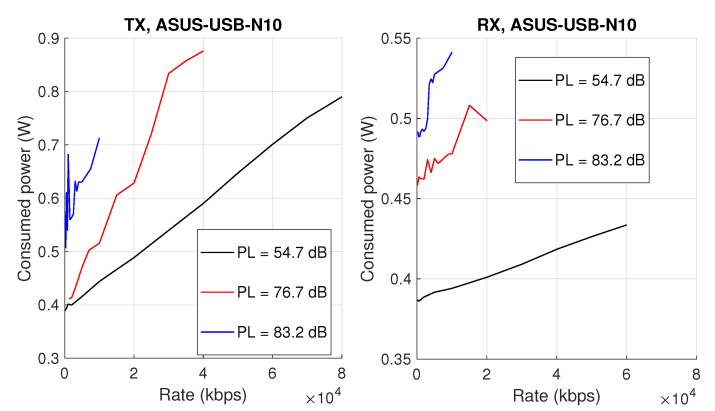
Comparison of measured power consumption for a single transceiver in TX/RX mode.

During the experiment, three different static (with no mobility) settings were considered: first, where both devices are located in the same room; second, where they are placed in neighboring rooms (one wall between transmitter and receiver); and third, where the two wirelessly connected computers are in non-adjacent rooms (i.e., two walls and one room between transmitter and receiver). The measured pathlosses corresponding to these three scenarios were: 54.7 dB, 76.7 dB, and 83.2 dB, respectively. These values were measured with a signal generator, spectrum analyzer, and omnidirectional antennas. Selected detailed results of certain measurements for a single device are shown in Figure 2 for 54.7 dB, 76.7 dB, and 83.2 dB pathloss values. We started collecting samples when the connection was established and active transmission was ongoing. A single measurement consisted of around 750 kilo samples (15 s, 50 kilo samples per second).

## 4. Power Modeling

After the power measurements and the processing of collected data, we started creation of the power consumption model. Model fitting, statistical processing, and plots creation were done using Matlab. First, after the connection of the WiFi dongle to the computer, with no connection to any Access Point (AP) set, the model is in an idle state with power consumption modeled as a single positive number D. After association to the AP it is expected that the minimum power in the case of no IP transmission (no traffic in Layer 3) will be higher that D. The overhead will be denoted in our model by *C* being a non-negative number. When transmission begins the power increase is expected to be proportional to the transmission rate [7,8,9]. The power increase depends on the operation mode (receiving or transmitting mode). Typically, the transmitter (TX) mode power consumption will be dominated by the power consumed by a high-power power amplifier. On the receiver (RX) side, the reception algorithms appear to be the most power consuming. As such, the TX and RX rate’s influence on the consumed power is modeled separately. The utilized model for a single pathloss value is [4]:

(1) $$P=AR_{\mathrm{TX}}+BR_{\mathrm{RX}}+C+D,$$

where *A* and *B* represent the transceiver-specific, positive coefficients for TX rate $R_{\mathrm{TX}}$ (in kbps) and RX rate $R_{\mathrm{RX}}$ (in kbps), respectively, and *P* is the total consumed power in Watts.

The above modeling was done for the shortest path between TX and RX, as this guarantees the smallest wireless channel variation and smoothest power consumption characteristics, as visible in Figure 2. Next, the model needs to be updated in order to consider the pathloss between the AP and WiFi dongle. Thus, in addition to the previously used measurements’ results for short distance communications, two additional setups were used: when the AP was distanced by 76.7 dB (behind one wall) and by 83.2 dB (behind two walls). The balance between model complexity (number of coefficients) and accuracy (measured by coefficient of determination) was obtained by the following power consumption model [4]:

(2) $$\begin{array}{cc} P= & A(1+E(\Gamma -54.7))R_{\mathrm{TX}}+B(1+F(\Gamma -54.7))R_{\mathrm{RX}}\\ & +C+D+(\Gamma -54.7)(Gsgn(R_{\mathrm{TX}})+Hsgn(R_{\mathrm{RX}})). \end{array}$$

**Figure 3:**
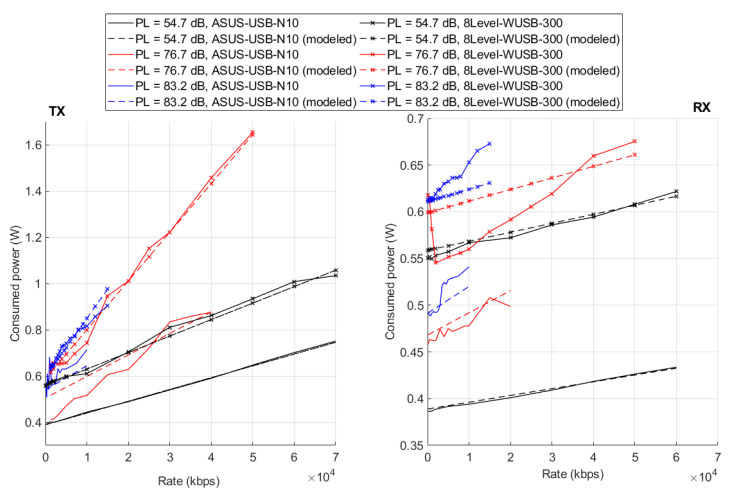
Comparison of measured and modeled power consumption for two selected transceivers in TX/RX mode.

In the formula, sgn() denotes the signum function, and Γ—the pathloss in dB between WiFi dongle and AP. Moreover, new, device-specific, coefficients E,F,G,H are included. Next, it is assumed that the parameters A,B,C,D are known for each WiFi dongle after the previous stage; thus only the values of E,F,G, and *H* have to be estimated. It is reasonable to assume that the consumed power increases with increasing pathloss, and the rationale behind this linear dependence in dB scale is derived in the Appendix A. The parameters were optimized in order to minimize mean squared error using the interior-point method. The resultant coefficients are shown in Table 2 along with mean values and standard deviation. Power consumption is compared for two selected transceivers in Figure 3. While the model can quite reliably approximate the power consumption of each device, there was a significant difference in the power consumption between different devices.

## 5. Stochastic Power Consumption Model

It was noted in the previous section that the power consumption depends not only on the the utilized chipset but also on the vendor-specific design. As such these 14 measured devices can be treated as samples from the population of all existing WiFi dongles. Statistical modeling was carried out to find the most suitable distributions for all of the model’s coefficients. It was found that A and B can be modeled as squared log-normal variables, D as a log-normal random variable, C, E, F, and G as squared normal random variables, and G and H as absolute values of normal random variables. Additionally, these normal variables have to be scaled and correlated considering model coefficients’ dependence.

Let us denote the 14×8 matrix of all coefficients as:

(3) $$M=[\ln(\sqrt{A})\;\ln(\sqrt{B})\;\sqrt{C}\;\ln(D)\;\sqrt{E}\;\sqrt{F}\;G\;H]$$

Each of the constituting vertical vectors contains a given model parameter for 14 measured devices. A vector composed of mean values of all modified coefficients is:

(4) $$\begin{array}{cc} \mu = & [E[\ln(\sqrt{A})]\;E[\ln(\sqrt{B})]\;E[\sqrt{C}]\;E[\ln(D)]\\ & E[\sqrt{E}]\;E[\sqrt{F}]\;E[G]\;E[H]] \end{array}$$

where E[ ] denotes the expectation over the 14 available samples. Additionally, the covariance matrix of these variables is Σ=cov(M). The covariance matrix is a positive semidefinite matrix so that Cholesky decomposition can be calculated giving $\Sigma =K^{T}K$.

Generation of a single set of random coefficients starts from the generation of eight independent, standard normal random variable samples, which are stored in a vector z. The vector of correlated and scaled normal random variables y is:

(5) $$y=K^{T}z+\mu^{T}.$$

**Figure 4:**
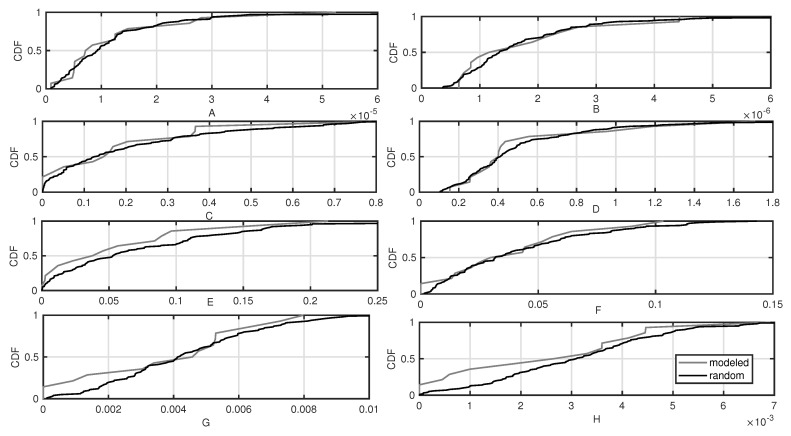
Cumulative distribution functions of power consumption mode coefficients: measurement vs. stochastic distribution.

Finally, random variables representing model parameters are: $A={\left(\exp\left(y_{1}\right)\right)}^{2},\;B={\left(\exp\left(y_{2}\right)\right)}^{2},\;C=y_{3}^{2},\;D=\exp\left(y_{4}\right),\;E=y_{5}^{2},\;F=y_{6}^{2},\;G=|y_{7}\left|,\;H=\right|y_{8}|.$ The coefficients that resulted from the measurements and modeling (“modeled”) were compared with 1000 coefficients generated randomly according to the scheme described above (“random”). In Figure 4, the cumulative distribution functions (CDFs) of the coefficients are shown. It is visible that the assumed distributions quite reliably reflect the empirical data distributions.

## 6. Model Verification

**Figure 5:**
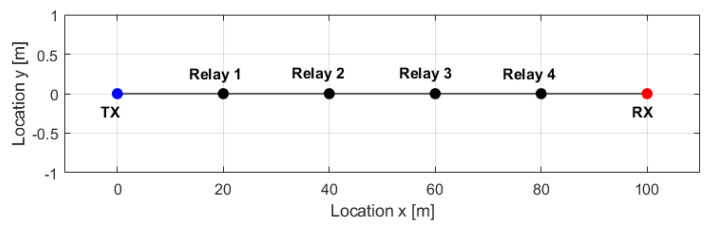
Example of network topology used in simulations (case with four relays).

In order to check the usefulness of the proposed energy consumption model, we conducted computer simulations. The simulation scenario assumes that there is a transmitter that sends data at a constant rate to the receiver. Between them there can be relays. A line topology is assumed, as presented in Figure 5. The frequency was set to 2.4 GHz, bitrate to 1 Mbps, and the pathloss model was WINNER-II (A1 scenario—in building—with line-of-sight propagation) [26]. In such a multi-transceiver network, a single device power consumption is typically unknown. In addition it can vary as a result of varying market shares of a given vendor or even single transceivers. The proposed stochastic energy consumption model allows to characterize the resultant power consumption as a random variable. The distance between the first transmitter and the last receiver was fixed to 100 m. Each transceiver’s parameters were randomly generated according to the procedure presented in Section 5 in 100,000 random trials.

**Figure 6:**
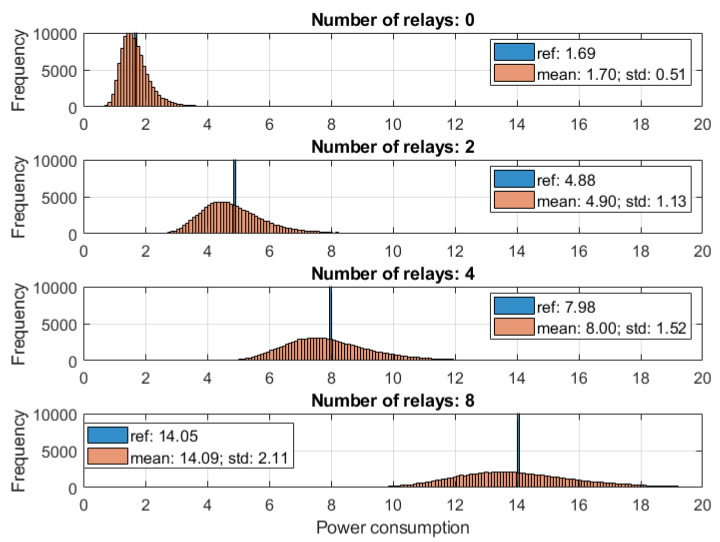
Energy consumption through 100,000 iterations with random transceiver parameters generation.

It was assumed that 0, 2, 4, or 8 relays are placed in between the transmitter and the receiver, equally distanced to the closest nodes. The power consumption histogram is compared in Figure 6 for the proposed model and a reference solution (*ref* in the figure). The reference model assumes that each of the devices (transmitter, receiver, and each of the relays) has the same *mean* power consumption characteristics, i.e., values of coefficients from the row “Mean” in Table 2 were used. It is shown that the energy consumption distribution gave much more information than a single value obtained by a reference model. The number of relays influenced not only the distribution variance but also its shape.

## 7. Conclusions

Measuring the power consumption of a number of real devices allowed us to create a unified energy consumption model. The model can be successfully used when designing a wireless network to faithfully reproduce the variability of characteristics of real devices. The coefficients presented in the paper are valid for IEEE 802.11 g transceivers with standard configuration in an office environment; however the proposed concept is generic and can be used for other communication standards as well. It can be very useful when characterizing the energy consumption of a heterogeneous wireless network on a system level. The future steps can be measurements and modeling of power consumption of 802.11g transceivers while changing their setup or in a different environment. While qualitatively the proposed model is expected to be valid, the model parameters’ values will have to be changed. It is also possible to extend the proposed model to another wireless communication standard, e.g., Bluetooth.

## Figures and Tables

**Table 1:** The set of measured wireless transceivers [4].

Manufacturer	Name	Applied Chipset
8LEVEL	WUSB-300	RTL8192CU
ASUS	USB-N10	RTL8188CUS
	USB-N14	RT5372
D-LINK	DWA-125	RT3070L
	DWA-140	RT5372L
Edimax	EW-7811U	RTL8188CUS
LINKSYS	WUSB54GC	RT2571W/RT2528
	AE1200	BCM43235
MODECOM	MC-UN11A2	RT5370N
NETGEAR	WNA3100	BCM43231
TP-LINK	TL-WN422G	AR9002U/AR9271
	TL-WN822N	RTL8192CU
	TL-WN823N	RTL8192CU
	TL-WN727N	MT7601U

**Table 2:** Modeling results [4].

Manufacturer	Model	*A*	*B*	*C*	*D*	*E*	*F*	*G*	*H*
8Level	WUSB-300	$7.1\times 10^{-6}$	$9.6\times 10^{-7}$	0.204	0.355	0.089	0.013	0.0014	0.0018
ASUS	USB-N10	$5.1\times 10^{-6}$	$7.3\times 10^{-7}$	0.0258	0.363	0.038	0.103	0.00528	0.0036
	USB-N14	$1.3\times 10^{-5}$	$1.5\times 10^{-6}$	0.353	0.4	0.023	0.03	0.0073	0.00045
D-Link	DWA-125	$8.8\times 10^{-7}$	$2.1\times 10^{-6}$	0.16	1.19	0.046	0.043	$1.1\times 10^{-8}$	$1.7\times 10^{-9}$
	DWA-140	$1.3\times 10^{-5}$	$1.9\times 10^{-6}$	0.365	0.437	0.0026	$7.5\times 10^{-9}$	0.0034	0.00059
EDIMAX	EW-7811U	$5\times 10^{-6}$	$6.6\times 10^{-7}$	$1.1\times 10^{-11}$	0.41	0.012	0.044	0.0053	0.0041
LINKSYS	AE1200	$5.2\times 10^{-5}$	$4.4\times 10^{-6}$	0.82	0.15	0.0074	0.09	0.0048	0.0036
	WUSB54GC	$1.5\times 10^{-5}$	$2.4\times 10^{-6}$	$4.4\times 10^{-13}$	1.71	0.057	$2.3\times 10^{-11}$	$3.2\times 10^{-15}$	$2.8\times 10^{-14}$
MODECOM	MC-UN11A2	$2.8\times 10^{-5}$	$8.4\times 10^{-7}$	0.17	0.26	$1.7\times 10^{-10}$	0.051	0.00091	0.0033
NETGEAR	WNA3100	$8.4\times 10^{-6}$	$4.4\times 10^{-6}$	0.051	0.96	0.21	0.015	0.008	0.001
TP-LINK	TL-WN422G	$2.6\times 10^{-5}$	$2.7\times 10^{-6}$	0.367	0.31	0.0022	0.021	0.0052	0.0045
	TL-WN727N	$4.7\times 10^{-6}$	$6.4\times 10^{-7}$	0.12	0.26	0.096	0.056	0.003	0.0026
	TL-WN822N	$5.2\times 10^{-6}$	$8.4\times 10^{-7}$	$5.9\times 10^{-8}$	0.56	0.084	0.025	0.0046	0.0045
	TL-WN823N	$6.9\times 10^{-6}$	$1.2\times 10^{-6}$	0.15	0.4	0.15	0.064	0.0063	0.0069
Mean		$1.4\times 10^{-5}$	$1.8\times 10^{-6}$	0.20	0.55	0.059	0.04	0.004	0.0026
Standard deviation		$1.4\times 10^{-5}$	$1.3\times 10^{-6}$	0.22	0.43	0.064	0.03	0.003	0.0021

## Appendix A

This section is to show that the consumed power can be proportional to pathloss in dB. Let us assume that the achievable rate *R* is:

(A1) $$R=B\cdot \log_{2}(1+\frac{P_{T}10^{-\frac{\Gamma_{0}}{10}}}{N})$$

where the pathloss equals $\Gamma_{0}$ (in dB), the transmit power is $P_{T}$, *B* denotes system bandwidth and *N* is noise plus interference power at the receiver. If the pathloss Γ is smaller than $\Gamma_{0}$, the same mean rate can be achieved in only part *x* of the available time (where x∈(0;1)). This is the situation with the IEEE 802.11 protocol, which utilizes the adaptation of modulation and coding schemes to minimize transmission time, thus reducing the probability of packet collision and maximizing resources available for other transmitters. Over the 1−x part of time there is no transmission. In this case the same rate can be obtained as:

(A2) $$R=xB\cdot \log_{2}(1+\frac{P_{T}10^{-\frac{\Gamma }{10}}}{N}).$$

Knowing that both rates are equal, both equations can be merged, giving:

(A3) $$1+\frac{P_{T}10^{-\frac{\Gamma_{0}}{10}}}{N}={\left(1+\frac{P_{T}10^{-\frac{\Gamma }{10}}}{N}\right)}^{x}.$$

Assuming that the signal-to-noise power ratio, i.e., $\frac{P_{T}10^{-\frac{\Gamma_{0}}{10}}}{N}$, is much higher than 1, the unity on both sides can be omitted, giving:

(A4) $$\frac{P_{T}10^{-\frac{\Gamma_{0}}{10}}}{N}={\left(\frac{P_{T}10^{-\frac{\Gamma }{10}}}{N}\right)}^{x},$$

(A5) $${\left(\frac{P_{T}}{N}\right)}^{1-x}=10^{-\frac{\Gamma x}{10}}10^{\frac{\Gamma_{0}}{10}}=10^{-\frac{\Gamma x-\Gamma_{0}}{10}}.$$

With a modicum of algebra we obtain:

(A6) $$x=\frac{\Gamma_{0}-10\log_{10}(\frac{P_{T}}{N})}{\Gamma -10\log_{10}(\frac{P_{T}}{N})}=\frac{1-\frac{\Gamma_{0}}{10\log_{10}(\frac{P_{T}}{N})}}{1-\frac{\Gamma }{10\log_{10}(\frac{P_{T}}{N})}}.$$

Utilizing the previous assumption that $\frac{P_{T}10^{-\frac{\Gamma_{0}}{10}}}{N}>1$ and observing that $\frac{P_{T}10^{-\frac{\Gamma }{10}}}{N}>\frac{P_{T}10^{-\frac{\Gamma_{0}}{10}}}{N}$, it can be obtained that $\frac{\Gamma }{10\log_{10}(\frac{P_{T}}{N})}<1$. As such, the Taylor series expansion of $\frac{a}{1-x}$ for small *x* can be used. Knowing that $\frac{a}{1-x}=a\sum_{n=0}^{\infty }x^{n}$, the two first components can be used to approximate (A6) as:

(A7) $$x\approx a+a\frac{\Gamma }{10\log_{10}(\frac{P_{T}}{N})}$$

where $a=\frac{\Gamma_{0}}{10\log_{10}(\frac{P_{T}}{N})}$.

It is reasonable to assume that in a given environment, for a given modulation and coding scheme the consumed power will be fixed while transmitting, e.g., $P_{MAX}$. If the transmission is carried only by *x* part of the time, then the mean consumed power will be close to $xP_{MAX}$. As such mean consumed power is proportional to pathloss in dB, i.e., const+constΓ.
